# Serum osteoprotegerin in prevalent hemodialysis patients: associations with mortality, atherosclerosis and cardiac function

**DOI:** 10.1186/s12882-017-0701-8

**Published:** 2017-09-07

**Authors:** Sílvia Collado, Elisabeth Coll, Carlos Nicolau, Manel Azqueta, Mercedes Pons, Josep M Cruzado, Bernat de la Torre, Ramón Deulofeu, Sergi Mojal, Julio Pascual, Aleix Cases

**Affiliations:** 1 Nephrology Department Hospital del Mar, Institut Hospital del Mar d’Investigacions Mèdiques, C/ Paseo Marítimo, 25-29, 08003 Barcelona, Spain; 2 Nephrology Department, Fundació Puigvert, Barcelona, Spain; 3 CDI, Hospital Clínic, Barcelona, Spain; 4 Cardiology Department, Hospital Clínic, Barcelona, Spain; 5 CETIRSA Barcelona, Fresenius Medical Care, Barcelona, Spain; 6 Institut Hemodiàlisi Barcelona, Diaverum, Barcelona, Spain; 7 CD Palau, Diaverum, Barcelona, Spain; 8 CDB, Hospital Clínic, Barcelona, Spain; 9 Department of Statistics, Institut Mar D’Investigacions Mèdiques, Barcelona, Spain; 10 Universitat Autónoma Barcelona and Universitat Pompeu Fabra. Nephropathies Research Group Coordinator, Institute Mar for Medical Research, Barcelona, Spain; 11 Nephrology Department, Hospital Clínic, Universitat de Barcelona, IDIBAPS, Barcelona, Spain

**Keywords:** Cardiovascular disease, Cardiac dysfunction, Fetuin-a, Hemodialysis, Mortality, Osteoprotegerin

## Abstract

**Background:**

To assess whether serum osteoprotegerin (OPG) and/or fetuin-A predict mortality and cardiovascular (CV) morbidity and mortality in hemodialysis patients.

**Methods:**

Multicenter, observational, prospective study that included 220 hemodialysis patients followed up for up to 6 years. Serum OPG and fetuin-A levels were measured at baseline and their possible association with clinical characteristics, CV risk biomarkers, carotid ultrasonographic findings, as well as their association with overall and CV mortality and CV events were assessed.

**Results:**

During a mean follow-up of 3.22 ± 1.91 years, there were 74 deaths (33.6%) and 86 new cardiovascular events. In the Kaplan-Meier survival analysis, the highest tertile of OPG levels was associated with higher overall mortality (*p* = 0.005), as well as a higher, although non-significant, incidence of CV events and CV mortality. In contrast, fetuin-A levels did not predict any of these events. OPG levels were directly associated with age, the Charlson comorbidity index (CCI), prevalent cardiovascular disease, carotid intima-media thickness, adiponectin, troponin-I and brain natriuretic peptide (BNP). OPG showed a negative correlation with left ventricular ejection fraction (LVEF) and phosphate levels. In the multivariate Cox proportional hazard analysis, all-cause mortality was associated with the highest tertile of OPG (HR:1.957, *p* = 0.018), age (HR:1.031, *p* = 0.036), smoking history (HR:2.122, *p* = 0.005), the CCI (HR:1.254, *p* = 0.004), troponin-I (HR:3.894, *p* = 0.042), IL-18 (HR:1.061, *p* < 0.001) and albumin levels (HR:0.886, *p* < 0.001). In the bootstrapping Cox regression analysis, the best cut-off value of OPG associated with mortality was 17.69 pmol/L (95%CI: 5.1–18.02).

**Conclusions:**

OPG, but not fetuin-A levels, are independently associated with overall mortality, as well as clinical and subclinical atherosclerosis and cardiac function, in prevalent hemodialysis patients.

**Electronic supplementary material:**

The online version of this article (10.1186/s12882-017-0701-8) contains supplementary material, which is available to authorized users.

## Background

Overall mortality remains exceedingly high in hemodialysis patients, mainly due to cardiovascular (CV) disease [1]. The higher prevalence of classical CV risk factors do not fully explain the excess CV risk in this population, and the Framingham risk score poorly predicts CV events in end-stage renal disease (ESRD) [2]. In recent years, the possible contributory role of new and uraemia-related CV risk factors has been thoroughly investigated in this population. ESRD results in the accumulation of uremic toxins, volume overload, electrolyte abnormalities, metabolic acidosis, inflammation, anaemia, bone-mineral disorders (BMD), oxidative and carbamyl stress, and neurohumoral or metabolic abnormalities that can contribute to the excess mortality in these patients [3]. The role of chronic kidney disease (CKD)-associated BMD and vascular calcification in this increased mortality risk in ESRD is also a matter of intensive research. Vascular calcification is a common finding in ESRD, appears earlier and progresses faster than in the general population, and it is associated with a higher mortality risk [4]. Several CKD-BMD biomarkers, such as fetuin-A, osteoprotegerin (OPG), osteopontin, fibroblast growth factor-23 (FGF-23), receptor activator of nuclear factor-κB ligand (RANKL), klotho or bone morphogenetic protein 7 (BMP-7) have been associated with vascular calcification [5, 6].

OPG is a soluble cytokine of the tumour necrosis factor (TNF) receptor superfamily expressed in osteoblasts, but also in endothelial cells (EC), vascular smooth muscle cells (VSMC), or in the heart; and it is modulated by inflammatory cytokines. It acts as a decoy receptor for RANKL, thus inhibiting osteoclast differentiation and activity; and also binds TNF-related apoptosis-inducing ligand (TRAIL), preventing TRAIL-induced apoptosis [7]. Although OPG protects against vascular calcification, patients with ESRD show increased serum OPG levels and in different studies, it has been directly associated with vascular calcification [8], arterial stiffness [9, 10] and increased mortality risk in this population [11].

Fetuin-A is a circulating glycoprotein secreted by the hepatocytes, which acts as a potent systemic inhibitor of calcification and a negative acute phase reactant. It prevents calcium and phosphate precipitation in serum, and protects from arterial media calcification by inhibiting VSMC apoptosis and preventing basic calcium particle nucleation in the extracellular matrix. In hemodialysis patients, its plasma levels are lower than in healthy controls [12]. Low fetuin-A levels have been associated with severe and extensive vascular calcification, arterial stiffness [9], as well as with increased all-cause and cardiovascular mortality in this population in some studies [11, 12], but not all [13].

In this study we have evaluated the possible relationship of serum OPG and fetuin-A levels with overall mortality risk and cardiovascular morbidity and mortality in a prevalent population of hemodialysis patients, as well as their association with atherosclerosis and/or vascular calcification, as well as with cardiac structure and function.

## Methods

### Design

This was a multicenter, observational, prospective study that included 220 patients with ESRD on maintenance hemodialysis for at least 6 months from a University Hospital and 4 satellite dialysis units. Patients had to be clinically stable and without evidence of clinical heart failure at the time of enrolment. No other specific exclusion criteria were applied. The study was conducted in accordance with the Declaration of Helsinki and approved by the local Ethics Committee. Patients agreed to participate in the study and signed an informed written consent.

All patients were receiving conventional hemodialysis, 3.5 to 4.5 h, three times weekly, with dialyzers of cellulose diacetate or polysulphone membranes, and a bicarbonate dialysate. Low molecular weight heparin was used as standard anticoagulation. Dialysis prescription was guided to achieve a target Kt/V ≥ 1.3.

In a subgroup of 101 (45.9%) unselected patients, we performed a carotid Doppler ultrasound study, while in 156 (70.9%) unselected patients an echocardiographic study was performed.

Demographic, anthropometric, clinical and biochemical data were collected at baseline, including: age, sex, time on hemodialysis, previous kidney transplants and aetiology of renal disease; history of cardiovascular risk factors (hypertension, diabetes, hypercholesterolemia and smoking); patients’ comorbidities, characteristics of the hemodialysis sessions, and concomitant treatments (antihypertensive, lipid-lowering, antiplatelet/anticoagulant agents, vitamin D analogues, erythropoiesis-stimulating agents and doses of calcium salts, such as acetate or carbonate, as daily dose of calcium in g/day; and intravenous iron).

Attending physicians periodically evaluated the participating patients in their dialysis units for up to 6 years. Death and cause, kidney transplantation or transfers to another dialysis unit were recorded. Clinical events were also registered, including cardiovascular events of cardiac origin (defined as coronary heart disease events, congestive heart failure or cardiac arrhythmias), cerebrovascular events (stroke or transient ischemic attack) and peripheral ischemic events (peripheral artery disease events, mesenteric ischemia, etc.). Patients were followed until death, loss to follow-up or kidney transplantation. All clinical events recorded were evaluated and reviewed by SC, EC and AC before being assigned.

### Methods

Blood pressure was measured before each hemodialysis session during one week and the mean of all three sessions was considered. Blood samples were obtained before the second dialysis session of the week after 20–30 min of rest in the supine position. Routine biochemical and haematological parameters were measured at baseline in the laboratory facilities of our institution using pertinent autoanalyzers: Blood urea nitrogen (BUN), creatinine, calcium, phosphate, total cholesterol, HDL-cholesterol, triglycerides, lipoprotein (a) haemoglobin, fibrinogen, ferritin, or C reactive protein (CRP). LDL cholesterol was calculated by the Friedwald’s formula. Homocysteine was determined by immunoassay in an Advia-Centaur autoanalyzer (Siemens Spain, Barcelona), using reagents from the manufacturer. Troponin-I, brain natriuretic peptide (BNP) and iPTH were measured by electrochemiluminescence assay in an Advia Centaur analyzer (Siemens, Tarrytown, USA) using Siemens reagents. Additional special tests included: OPG, measured through an ELISA kit (Biomedica Medizinprodukte GmbH, Vienna, Austria), fetuin-A, by an ELISA kit supplied by Epitope Diagnostics (Epitope Diagnostics, San Diego, California, USA), asymmetric dimethylarginine (ADMA) by HPLC, advanced oxidation protein products (AOPP), by a photometric Chloramine T method, malondialdehyde (MDA) by HPLC measurement of thiobarbituric acid reaction products, interleukin 6 (IL-6) and IL-10 by enzyme immunoassay from Diasource (Louvain-la-Neuve, Belgium), IL-18 by MBL (Nagoya, Japan) and adiponectin by radioimmunoassay (Millipore Corp. Billerica, MA, USA).

Carotid Doppler ultrasound was performed with a colour Doppler ultrasound equipment and software to measure IMT and evaluation of plaques. Carotid IMT was measured at 1 cm prebifurcation, explored in a longitudinal section on the far wall, obtaining 4 measurements at regular intervals. A value of an IMT ≤ 0.9 mm was considered as normal, according to the criteria of the European Guidelines for the management of Hypertension of the ESC-ESH of 2013 [14]. The presence of plaque was defined as a focal structure that invaded the arterial lumen of at least 0.5 mm or >50% of the surrounding IMT or demonstrated a thickening >1.5 mm measured from the media-adventitia interface to the intima-lumen interface [15]. Carotid atherosclerotic disease (CAD) was classified in 4 degrees of severity (Grade 1: IMT <0.9 mm, grade 2: IMT > 0.9 mm, grade 3: presence of carotid plaques with stenosis <50% and grade 4: presence of carotid plaques with stenosis >50%). Grades 3 and 4 were considered severe degrees.

The echocardiographic studies were performed with a commercially available system (VIVID 7, General Electrics; Milwaukee, WI and Sonos 5500 Philips, Andover, MA, USA). Measurements included left ventricular ejection fraction (LVEF) and left ventricular wall mass indexed (LVMI) to body surface area; LV was calculated from M-mode recording, according to the recommendations of the American Society of Echocardiography [16].

### Statistical analysis

Results are expressed as mean ± standard deviation, median and interquartile range, or as hazard ratio (HR) with its corresponding 95% confidence interval (95%CI), as appropriate. The Kolmogorov-Smirnov test was applied to continuous variables to check for distribution normality. The Student’s t-test for independent samples and the Mann-Whitney U test were used for comparative statistics, where appropriate. The chi square test was used for qualitative variables. The Spearman rank correlation coefficient, was used when at least one variable was not assumed to be normally distributed. Survival analysis was carried out by using the Kaplan-Meier test and the multivariate Cox regression analysis. Intervals and formal test for the proportional hazards assumption of the OPG risk prediction model was performed by bootstrapping (95%CI). Significance was set at *p* < 0.05. Data were analysed by using the SPSS software (Statistical Package for Social Sciences, version 22.0, SPSS Inc).

## Results

### Demographics and baseline assessment

We included 220 patients followed-up between 2004 and 2010; 70 % were male, with a mean age of 61.1 ± 6.1 years, and median time on hemodialysis of 31 months [interquartile range: 9–77.7]. The mean Charlson comorbidity index was 5.76 ± 2.46 points, and 23.2% of the patients had a previous kidney transplant. Their demographic parameters and the prevalence of classical CV risk factors are summarized in Table 1. The most common causes of ESRD were vascular and glomerular (20% and 17.3% respectively), followed by diabetic nephropathy (16.8%) and unknown aetiology (16.4%). The prevalence of CV disease at baseline was 54.5%: 47.7% of patients had a history of cardiac disease and 24.1% had a history of vascular disease (cerebrovascular disease or peripheral artery disease).

**Table 1:** Descriptive analysis of the hemodialysis population

Patients (*n*)	220
Age (years)	61.1 ± 6.1
Sex (male/female)	154 (70%) / 66 (30%)
Charlson Comorbidity Index	5.76 ± 2.46
Body mass index (BMI) (Kg/m^2^)	24.33 ± 4.39
Time on HD (months) (median)	31 [9, 77.7]
Previous kidney transplant	51 (23.2%)
Smoking (yes/no/former)	35/130/55 (15.9%/59.1%/25%)
Diabetes mellitus (yes/no)	56 (25.5%)/164 (74.5%)
Hypertension (yes/no)	194 (88.2%)/26(11.8%)
Dyslipidaemia (yes/no)	84 (38.2%)/136 (61.8%)
IMT, mean (mm)	0.789 ± 0.282
IMT > 0.9 mm	35 (15.9%)
Presence of Plaques	85 (84.2%)
Calcified Plaques	69 (68.4%)
Presence of Stenosis	40 (72.3%)
LVH (%)	54 (24.5%)
LVMI (g/m^2^)	96.27 ± 26.07
LV ejection fraction (%)	58.34 ± 10.73

Data are shown as mean ± SD or cases (percentage) unless otherwise specified

The median serum level of OPG was 8.78 pmol/L [interquartile range: 6.07–12.95] and of fetuin-A 36.88 ng/ml [interquartile range: 26.51–51.46]. The associations of OPG and fetuin with qualitative variables (Man-Whitney U test) and with quantitative variables (Spearman’s rank correlation coefficient) are described in Table 2A and B, respectively. OPG levels were associated with age, prevalent arrhythmia, the Charlson comorbidity index, levels of troponin-I, BNP or adiponectin, beta-blocker treatment, phosphate, Ca x P product, or creatinine levels. No association was observed between OPG levels and fetuin-A or concomitant treatments for BMD (vitamin-D analogues or dose of calcium salts). Fetuin-A levels were associated with age, diabetes mellitus, and a previous kidney transplant, the Charlson comorbidity index, levels of IL-6, homocysteine, ADMA, or malondialdehyde and the daily dose of calcium salts. No correlation was found with calcium, phosphorus, iPTH or other concomitant treatments, such as vitamin D analogues (Table 2).

**Table 2:** Associations of osteoprotegerin and fetuin-A in the univariate analysis

A) Mann-Whitney U-test				
	Osteoprotegerin		Fetuin-A	
Variable	Median (interquartile range)	*P*-Value	Median (interquartile range)	*P*-Value
Previous Transplant				
Yes	9.77 (5.14–14.27)	0.944	44.1 (30.18–58.51)	**0.022**
No	8.58 (6.21–12.69)		35.24 (25.55–49.83)	
Diabetes Mellitus				
Yes	9.29 (7.06–11.76)	0.575	31.12 (20.8–43.94)	**0.008**
No	8.63 (5.72–13.01)		37.46 (28.04–54.02)	
Left Ventricular Hypertrophy				
Yes	10.02 (7.6–12.88)	0.068	34.92 (26.13–51.19)	0.639
No	8.23 (5.65–12.99)		37.02 (26.93–51.97)	
Arrhythmia				
Yes	12.1 (6.89–15.1)	**0.009**	35.17 (26.79–55.41)	0.916
No	8.26 (5.91–11.85)		37.02 (26.50–50.89)	
Statin treatment				
Yes	7.93 (6.1–10.62)	0.086	35.17 (24.21–54.02)	0.631
No	9.67 (6.05–13.43)		37.02 (26.87–50.75)	
ACEI/ ARB treatment				
Yes	7.84 (5.03–11.30)	0.059	39.99 (33.68–57.52)	0.078
No	9.08 (6.38–13.04)		35.17 (26.11–50.89)	
Beta-blockers treatment				
Yes	7.27 (4.99–11.49)	**0.038**	37.03 (27.35–54.98)	0.920
No	9.08 (6.39–13.04)		36.58 (26.50–50.92)	
Carotid Plaques				
Yes	8.5 (6.1–12.69)	**0.002**	31.24 (23.48–44.91)	0.728
No	5.98 (3.41–7.77)		35.34 (22.37–51.71)	
Calcified Plaques				
Yes	8.22 (6.23–12.66)	**0.038**	30.14 (23.49–44.34)	0.378
No	6.94 (4.97–10.64)		37.04 (21.54–51.46)	
Severe Degrees of CAD				
Yes	8.5 (6.1–12.69)	**0.002**	31.24 (23.48–44.91)	0.728
No	5.98 (3.41–7.77)		35.34 (22.37–51.71)	
B) Spearman rank correlation test				
	Osteoprotegerin		Fetuin-A	
Variable	Rho Spearman	*P*-Value	Rho Spearman	*P*-Value
Age (years)	0.383	**< 0.001**	−0.161	**0.018**
LV mass (gr)	0.126	0.117	−0.19	**0.020**
LV ejection Fraction (%)	−0.206	**0.01**	−0.056	0.491
Charlson Comorbility Index	0.38	**< 0.001**	−0.14	**0.040**
Calcium (mg/dl)	0.115	0.088	−0.015	0.828
Phosphate (mg/dl)	−0.245	**< 0.001**	0.011	0.870
iPTH (pg/ml)	−0.118	0.083	−0.083	0.228
Ca-P product	−0.2	**0.003**	0.012	0.861
Dose of calcium salts (gr/day)	−0.016	0.816	0.207	**0.003**
Creatinine (mg/dl)	−0.224	**0.001**	0.096	0.161
BUN (mg/dl)	−0.156	**0.039**	0.017	0.824
Troponin I (ng/ml)	0.207	**0.003**	−0.071	0.312
BNP (pg/ml)	0.305	**< 0.001**	−0.091	0.182
Systolic Blood Pressure (mmHg)	−0.008	0.907	−0.017	0.805
Diastolic Blood Pressure (mmHg)	−0.081	0.243	−0.028	0.690
Mean Blood Pressure (mmHg)	−0.049	0.479	−0.025	0.727
Adiponectin (μg/ml)	0.237	**< 0.001**	−0.04	0.558
IL-6 (pg/ml)	0.075	0.291	−0.16	**0.027**
CRP (mg/dl)	0.062	0.362	−0.013	0.846
Malondialdehyde (mMol/L)	0.086	0.205	0.179	**0.009**
Homocysteine (μmol/L)	−0.021	0.757	−0.137	**0.047**
ADMA (μmol/L)	−0.034	0.625	0.143	**0.040**
Carotid Intima-media thickness (mm)	0.234	**0.034**	−0.071	0.529

Significant results (*p*<0.05) are in bold

The presence of carotid atherosclerotic disease, defined by a pathological IMT and/or presence of carotid plaques with stenosis, or calcified atherosclerotic plaques was associated with higher OPG levels, but not with fetuin-A levels (Table 2). With respect to cardiac parameters, fetuin-A was negatively associated with LVMI, while OPG levels were negatively associated with LVEF.

### Prospective study

During a mean follow-up of 3.2 ± 1.91 years, 74 deaths (33.6%) were recorded. The causes were of cardiovascular origin in 35 cases and non-CV in 39 cases. New cardiovascular events were diagnosed in 86 cases (65.1% cardiac and 34.9% vascular). Kidney transplants occurred in 68 (30.9%) patients and 13 (5.9%) patients were lost to follow-up. Among the cardiovascular causes of death, the most frequent were sudden death (37.1%), myocardial infarction (22.9%), and stroke (11.4%) (Additional file 1: Table S1). Among the non-cardiovascular causes of mortality, the most frequent were infectious diseases (46.2%), followed by cancer (15.4%) and bleeding (12.8%).

**Fig. 1:**
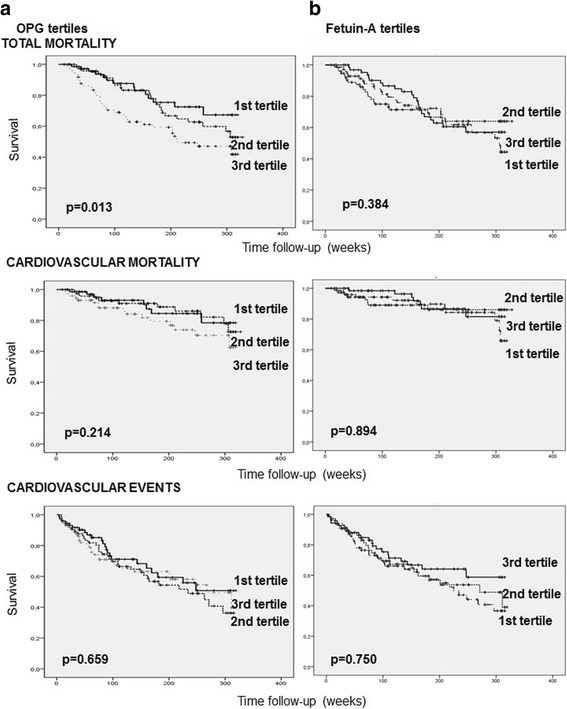
Event-free survival in patients according to osteoprotegerin (OPG) levels. **a** Total mortality, cardiovascular mortality and cardiovascular events comparing the three OPG serum level tertiles. **b** Total mortality, cardiovascular mortality and cardiovascular events comparing patients in the highest tertile of OPG levels with patients in the two other tertiles

In the Kaplan-Meier survival analysis, the highest OPG tertile (>11.44 pmol/L) was associated with a higher all-cause mortality (*p* = 0.005) compared to the first (<7.03 pmol/L) or second tertiles (7.03–11.44 pmol/L), as well as when comparing the third tertile vs the two other tertiles. Higher, although non-significant, incidence of cardiovascular events and cardiovascular mortality were also observed among patients in the third OPG tertile (Fig. 1). Fetuin-A levels distributed in tertiles, were not associated with any of these events.

### Cox regression analysis

In the univariate Cox regression analysis all-cause mortality, showed a positive relationship with age, left ventricular hypertrophy (LVH), prevalent cardiovascular disease, new CV events, the Charlson comorbidity index, smoking, and the highest tertile of serum OPG, troponin-I, BNP and CRP. A negative correlation was observed with a history of a previous kidney transplant, LVEF, diastolic blood pressure (BP), mean BP, serum albumin and creatinine. No correlation was found with calcium, phosphate, iPTH, fetuin-A levels neither its tertiles, nor with concomitant medications, such as daily dose of calcium salts or vitamin D analogues (Table 3).

**Table 3:** Univariate and Multivariate Cox proportional hazard analysis of overall mortality

	Univariate analysis		Multivariate analysis	
Variable	HR (CI 95%)	*P*-Value	HR (CI 95%)	*P*-Value
Age (years)	1.052 (1.032–1.072)	**<0.001**	1.031 (1.002–1.06)	**0.036**
Charlson Comorbidity Index	1.305 (1.194–1.427)	**<0.001**	1.254 (1.075–1.462)	**0.004**
Previous cardiovascular disease (yes/no)	2.185 (1.30–3.65)	**0.003**	-	
Incident cardiovascular disease (yes/no)	2.654 (1.646–4.279)	**<0.001**	-	
Left Ventricular Hypertrophy (yes/no)	1.764 (1.093–2.845)	**0.002**	-	
Previous Kidney Transplant (yes/no)	0.374 (0.186–0.751)	**0.006**	-	
Interdialytic weight gain (kg)	0.764 (0.579–1.007)	0.056	-	
Dyslipidaemia (yes/no)	1.255 (0.793–1.985)	0.332	-	
Diabetes Mellitus (yes/no)	1.401 (0.861–2.28)	0.174	-	
Hypertension (yes/no)	1.32 (0.606–2.878)	0.485	-	
Smoking (yes/no)	2.185 (1.376–3.47)	**0.001**	2.122 (1.251–3.599)	**0.005**
Systolic Blood Pressure (mmHg)	0.993 (0.983–1.004)	0.191	-	
Diastolic Blood Pressure (mmHg)	0.975 (0.957–0.994)	**0.010**	-	
Mean Blood Pressure (mmHg)	0.983 (0.968–0.999)	**0.032**	-	
Statin treatment (yes/no)	1.516 (0.929–2.473)	0.096	-	
Osteoprotegerin (highesttertile)	1.9 (1.203–2.999)	**0.006**	1.957 (1.123–3.411)	**0.018**
Calcium (mg/dl)	1.136 (0.9–1.434)	0.284	-	
Phosphate (mg/dl)	0.868 (0.745–1.013)	0.072	-	
iPTH (pg/ml)	0.999 (0.998–1.000)	0.114	-	
Fetuin-A (ng/ml)	0.994 (0.984–1.004)	0.230	-	
Albumin (g/l)	0.884 (0.844–0.925)	**<0.001**	0.886 (0.837–0.937)	**< 0.001**
C-reactive protein (mg/dl)	1.17 (1.09–1.252)	**<0.001**	-	
Adiponectin (μg/ml)	1.003 (0.973–1.033)	0.867	-	
IL-18 (pg/ml)	1.0 (1.00–1.001)	**0.011**	1.061 (1.036–1.087)	**<0.001**
Troponin I (ng/ml)	12.626 (3.795–42.01)	**<0.001**	3.894 (1.048–14.462)	**0.042**
BNP (pg/ml)	1.0 (1.0–1.001)	**0.001**	-	
LV ejection fraction (%)	0.966 (0.945–0.987)	**0.002**	-	

Significant results (*p*<0.05) are in bold

### Multivariate analysis

In the multivariate Cox proportional hazards ratio analysis all-cause mortality, was significantly associated with the highest tertile of OPG, age, smoking history, the Charlson comorbidity index, troponin-I, IL-18 and albumin levels (Table 3).

In the multivariate linear regression analysis serum OPG levels were independently associated with age, BNP levels, adiponectin levels and statin treatment (Table 4), while fetuin-A was only associated with malondialdehyde levels.

**Table 4:** Osteoprotegerin and Fetuin-A associations in the multiple linear regression analysis

Variable	β (CI 95%)	*P*-Value
**Multiple regression analysis OPG**		
Age (years)	0.113 (0.074–0.152)	**<0.001**
BNP (100 pg/ml)	0.202 (0.103–0.301)	**<0.001**
Adiponectin (μg/ml)	0.126 (0.042–0.210)	**0.003**
Statin Treatment (yes)	−1.977 (−3.386- -0.568)	**0.006**
**Multiple regression analysis Fetuin-A**		
Age (years)	−0.074 (−0.649–256)	0.392
Dose of calcium salts (gr/day)	0.063 (−0.003–0.007)	0.461
Malondialdehyde (mmol/L)	0.322 (0.211–0.699)	**<0.001**
IL-6 (pg/ml)	−0.154 (−0.118–0.007)	0.079

Adjusted R^2^ of model: 0.255

Significant results (*p*<0.05) are in bold

Regression models with OPG and Fetuin-A levels considered as continuous variables confirmed the robustness of the association.

The best OPG cut-off value with bootstrap validation in the Cox Proportional Hazards Ratio Model to predict overall mortality in our hemodialysis population was 17.69 pmol/L (95% CI: 5.1–18.02).

## Discussion

Serum OPG levels in the highest tertile were independently associated with overall mortality in prevalent hemodialysis patients. In contrast, serum levels of fetuin-A were not, thus suggesting that OPG may be a better prognostic biomarker in this population. Our results provide additional evidence on previous studies [17–19] and a recent metaanalysis [20] that reported an increased mortality risk associated with higher OPG levels. Furthermore, results from previous studies indicate that OPG levels could be predictive of fatal [18, 19] and non-fatal cardiovascular events in hemodialysis patients [21, 22]. Although elevated OPG levels have been associated with vascular calcification [8], and its progression [23], as well as with arterial stiffness [18, 19, 21] in ESRD patients, its predictive value on mortality is independent of the presence of arterial stiffness [10] or vascular calcification [24], thus suggesting that it may be involved in other pathways/mechanisms associated with increased mortality in this population, as discussed below.

OPG is expressed in vivo by EC and VSMC where it modulates apoptosis and inflammation. It is likely that OPG could play a role in the development of atherosclerosis by inhibiting TRAIL-induced apoptosis of vascular cells. In addition, circulating OPG may directly interact with heparan sulphates in the endothelium, favouring leukocyte adhesion [25]. In fact, the OPG/RANKL/RANK axis has been implied in several inflammatory responses and has also been associated with atherogenesis and endothelial dysfunction in non-CKD populations [25, 26]. In addition; OPG has been related to changes in vessel matrix composition, development of diabetic macrovascular disease, advanced atherosclerosis or plaque destabilization [27, 28]. However, it is not clear whether OPG is causally related to atherosclerosis or is a marker of the atherosclerotic burden, with high OPG levels being a compensatory response to subclinical cardiovascular disease, its progression; or of the transition of VSMC to an osteoblastic phenotype [11].

In this sense, we found that OPG levels were directly associated with carotid intima-media thickness, severe degrees of carotid atherosclerosis and calcified carotid plaques, as has been reported in the general population, and in agreement with a previous study [19], but not another [9]. However, despite that higher OPG levels have also been associated with an increased cardiovascular risk in the general population [29], therapeutic drugs targeting the RANK-RANKL-OPG axis have not been associated with an effect on cardiovascular events in randomized controlled trials in the general population.

We found intriguing the relationship of OPG with troponin-I (a marker of cardiac damage), BNP (a marker of biomechanical stress) and its inverse relationship with LVEF. Furthermore, BNP remained as an independent predictor of OPG levels in the multivariate analysis, in agreement with a previous study [19]. OPG mRNA is widely distributed in several organs, including the heart [30]. There is evidence of its intra-cardiac production and release into the coronary circulation, and it has been hypothesized that it may be involved in the development of LVH [31, 32]. Circulating OPG levels also reflect the severity of cardiovascular disorders, such as coronary artery disease, cardiac hypertrophy, and heart failure in the general population [33].

Two recent studies have shown an association of OPG with global longitudinal strain, a marker of subclinical LV dysfunction in diabetic hypertensive patients [34] and diabetic patients with heart failure [35]. Global longitudinal strain is a well validated and reproducible technique for the measurement of ventricular longitudinal deformation with predictive value regarding all-cause mortality, as well as a composite of cardiac death, malignant arrhythmia, hospitalization due to heart failure, urgent valve surgery or heart transplantation, and acute coronary ischemia [36]. Thus, we show for the first time an association between OPG levels and cardiac dysfunction in ESRD. It is tempting to speculate that the relationship between OPG and mortality in hemodialysis patients may be related, at least in part, to its putative association with the uremic cardiomyopathy, as has been proposed for the diabetic cardiomyopathy [34, 35].

OPG was not associated with diabetes or hypertension in our study. Although it has also been associated with markers of inflammation-malnutrition, we failed to find a relationship of OPG with CRP or with albumin, but the negative correlation observed with serum creatinine, BUN or phosphate levels might be attributed to its possible association with malnutrition. Actually, the relationship of this biomarker with CRP and/or albumin is not uniform in the literature [9]. OPG levels were not associated with CKD-BMD biomarkers in the multivariate analysis, in agreement with previous studies [22].

Fetuin-A is a known systemic inhibitor of vascular calcification. Low serum fetuin-A levels have been associated with all-cause and cardiovascular mortality in ESRD patients [11, 12, 37, 38], as well as with vascular calcification and arterial stiffness; although these associations have not been confirmed by others, both before, and after adjustment for several cardiovascular risk factors, or vascular calcification [24]. In our study, we failed to find an association between fetuin-A levels and all-cause, cardiovascular mortality or new cardiovascular events, or with carotid atherosclerosis, in agreement with previous studies [9]. Fetuin-A is a negative acute phase reactant, whose levels may be influenced by the presence of inflammation, as well as, insulin resistance, glucose intolerance, fatty liver disease, metabolic syndrome and an atherogenic lipid profile. In our study, we observed its association with diabetes mellitus, daily dose of calcium salts, left ventricular mass (but not with markers of cardiac dysfunction), and some biomarkers of inflammation or oxidative stress, such as IL-6, MDA or ADMA.

Apart from OPG levels, in our multivariate analysis overall mortality was also associated with age, the degree of comorbidity, and smoking, as expected; as well as with the levels of troponin-I (as a marker of cardiac damage) and IL-18 and albumin (as markers of inflammation-malnutrition) showing the complex pathogenesis of the disease in this population.

The limitations of the present study need to be addressed, including the relatively limited sample size and number of events. Thus, a larger patient cohort would be necessary to confirm our findings and to validate OPG as a biomarker of mortality in this population. Our analysis is limited to data obtained at baseline. The lack of repeated measurements of both OPG and fetuin-A levels during the follow-up, limited our ability to determine a causal relationship between OPG and mortality [39]. Furthermore, other markers of bone turnover were not measured (such as FGF23, sclerostin and Klotho). Another limitation is that we did not perform a carotid Doppler ultrasound and an echocardiographic study to the whole group, but to a subgroup of unselected patients. However, there are no reasons, to suspect significant bias in interpreting the results. Another limitation of the study was that we included patients with prevalent cardiovascular disease, which already have a higher cardiovascular risk. It would be interesting to repeat this study including patients without cardiovascular disease at baseline. Another potential limitation is the technical issues with the assay for the determination of OPG or fetuin-A (e.g. potential fragmentation) which are unexplored at present. In CKD, serum fetuin-A is mainly present as a fetuin-mineral complex (composed of fetuin-A, fibrinogen, fibronectin-I and calcium), and the fraction of total fetuin-A as a complex, rather than free fetuin-A increases progressively as the estimated glomerular filtration rate decreases [40].

## Conclusion

In conclusion, the present study shows that high levels of OPG, but not fetuin-A, are associated with increased mortality in prevalent hemodialysis patients. Higher levels of OPG are associated with both markers of subclinical atherosclerosis and cardiac function, but not with CKD-BMD biomarkers. Understanding the possible relationship between OPG and uremic cardiomyopathy deserves further studies.

## Additional file

Additional file 1: Table S1. Cardiovascular events and mortality during follow-up. (DOCX 14 kb)

## Abbreviations

ACEI: Angiotensin-converting enzyme inhibitors
ADMA: Asymmetric dimethylarginine
AOPP: Advanced oxidation protein products
ARB: angiotensin receptor blocker
BMD: Bone mineral disorders
BMI: Body mass index
BMP-7: Bone morphogenetic protein 7
BNP: Brain natriuretic peptide
BP: Blood pressure
BUN: blood urea nitrogen
Ca: Total calcium
CAD: Coronary artery disease
CKD: Chronic kidney disease
CRP: C-reactive protein
CV: Cardiovascular
EC: Endothelial cells
ESRD: End-stage renal disease
FGF-23: Fibroblast growth factor-23
HR: Hazard ratio
IL: Interleukin
IMT: Intima-media thickness
iPTH: Intact parathormone
LVEF: Left ventricular ejection fraction
LVH: Left ventricular hypertrophy
LVM: Left ventricular mass
LVMI: Left ventricular mass index
MDA: Malondialdehyde
OPG: Osteoprotegerin
Pi: Phosphate
RANKL: Receptor Activator of Nuclear Factor-κB ligand
TNF: Tumour necrosis factor
TRAIL: TNF-related apoptosis-inducing ligand
VSMC: Vascular smooth muscle cells

## References

[1] United States Renal Data System (2011). USRDS 2011 Annual Data Report: Atlas of Chronic Kidney Disease and End-Stage Renal Disease in the United States.

[2] Shah DS, Polkinghorne KR, Pellicano R, Kerr PG (2008). Are traditional risk factors valid for assessing cardiovascular risk in end-stage renal failure patients?. Nephrology.

[3] Park SH, Stenvinkel P, Lindholm B (2012). Cardiovascular biomarkers in chronic kidney disease. J Ren Nutr.

[4] Mizobuchi M, Towler D, Slatopolsky E (2009). Vascular calcification: the killer of patients with chronic kidney disease. J Am Soc Nephrol.

[5] Liabeuf S, Okazaki H, Desjardins L, Fliser D, Goldsmith D, Covic A, Wiecek A, Ortiz A, Martinez-Castelao A, Lindholm B, Suleymanlar G, Mallamaci F, Zoccali C, London G, Massy ZA (2014). Vascular calcification in chronic kidney disease: are biomarkers useful for probing the pathobiology and the health risks of this process in the clinical scenario?. Nephrol Dial Transplant.

[6] Moe SM, Chen NX (2008). Mechanisms of vascular calcification in chronic kidney disease. J Am Soc Nephrol.

[7] Schoppet M, Preissner KT, Hofbauer LC (2002). RANK ligand and osteoprotegerin: paracrine regulators of bone metabolism and vascular function. Arterioscler Thromb Vasc Biol.

[8] Nitta K, Akiba T, Uchida K, Kawashima A, Kawashima A, Yumura W, Kabaya T, Nihei H (2003). The progression of vascular calcification and serum osteoprotegerin levels in patients on long-term hemodialysis. Am J Kidney Dis.

[9] Pateinakis P, Papagianni A, Douma S, Efstratiadis G, Memmos D (2013). Associations of fetuin-a and osteoprotegerin with arterial stiffness and early atherosclerosis in chronic hemodialysis patients. BMC Nephrol.

[10] Speer G, Fekete BC, El Hadj OT, Szabó T, Egresits J, Fodor E, Kiss I, Logan AG, Nemcsik J, Szabó A, Németh ZK, Szathmári M, Tislér A (2008). Serum osteoprotegerin level, carotid-femoral pulse wave velocity and cardiovascular survival in haemodialysis patients. Nephrol Dial Transplant.

[11] Scialla JJ, Kao WH, Crainiceanu C, Sozio SM, Oberai PC, Shafi T, Coresh J, Powe NR, Plantinga LC, Jaar BG, Parekh RS (2014). Biomarkers of vascular calcification and mortality in patients with ESRD. Clin J Am Soc Nephrol.

[12] Ketteler M, Bongartz P, Westenfeld R, Wildberger JE, Mahnken AH, Böhm R, Metzger T, Wanner C, Jahnen-Dechent W, Floege J (2003). Association of low fetuin-a (AHSG) concentrations in serum with cardiovascular mortality in patients on dialysis: a cross-sectional study. Lancet.

[13] Ix JH, Shlipak MG, Sarnak MJ, Beck GJ, Greene T, Wang X, Kusek JW, Collins AJ, Levey AS, Menon V (2007). Fetuin-a is not associated with mortality in chronic kidney disease. Kidney Int.

[14] Mancia G, Fagard R, Narkiewicz K, Redon J, Zanchetti A, Böhm M, Christiaens T, Cifkova R, De Backer G, Dominiczak A (2013). 2013 ESH/ESC guidelines for the Management of Arterial Hypertension: the task force for the management of arterial hypertension of the European Society of Hypertension (ESH) and of the European Society of Cardiology (ESC). Eur Heart J.

[15] Touboul PJ, Hennerici MG, Meairs S, Adams H, Amarenco P, Bornstein N, Csiba L, Desvarieux M, Ebrahim S, Hernandez Hernandez R, Jaff M, Kownator S, Naqvi T, Prati P, Rundek T, Sitzer M, Schminke U, Tardif JC, Taylor A, Vicaut E, Woo KS (2012). Mannheim carotid intima-media thickness and plaque consensus (2004-2006-2011). An update on behalf of the advisory board of the 3rd, 4th and 5th watching the risk symposia, at the 13th, 15th and 20th European stroke conferences, Mannheim, Germany, 2004, Brussels, Belgium, 2006, and Hamburg, Germany, 2011. Cerebrovasc Dis.

[16] Schiller NB, Shah PM, Crawford M, DeMaria A, Devereux R, Feigenbaum H, Gutgesell H, Reichek N, Sahn D, Schnittger I (1989). Recommendations for quantitation of the left ventricle by two-dimensional echocardiography. American Society of Echocardiography Committee on standards, subcommittee on Quantitation of two-dimensional echocardiograms. J Am Society of Echocardiogr.

[17] Morena M, Terrier N, Jaussent I, Leray-Moragues H, Chalabi L, Rivory JP, Maurice F, Delcourt C, Cristol JP, Canaud B, Dupuy AM (2006). Plasma osteoprotegerin is associated with mortality in hemodialysis patients. J Am Soc Nephrol.

[18] Nakashima A, Carrero JJ, Qureshi AR, Hirai T, Takasugi N, Ueno T, Taniguchi Y, Lindholm B, Yorioka N (2011). Plasma osteoprotegerin, arterial stiffness, and mortality in normoalbuminemic Japanese hemodialysis patients. Osteoporos Int.

[19] Kuźniewski M, Fedak D, Dumnicka P, Stępień E, Kuśnierz-Cabala B, Cwynar M, Sułowicz W (2016). Osteoprotegerin and osteoprotegerin/TRAIL ratio are associated with cardiovascular dysfunction and mortality among patients with renal failure. Adv Med Sci.

[20] Pichler G, Haller MC, Kainz A, Wolf M, Redon J, Oberbauer R. Prognostic value of bone- and vascular-derived molecular biomarkers in hemodialysis and renal transplant patients: a systematic review and meta-analysis. Nephrol Dial Transplant. 2016; 10.1093/ndt/gfw387. 10.1093/ndt/gfw387 28025385

[21] Lee JE, Kim HJ, Moon SJ, Nam JS, Kim JK, Kim SK, Yun GY, Ha SK, Park HC (2013). Serum osteoprotegerin is associated with vascular stiffness and the onset of new cardiovascular events in hemodialysis patients. Korean J Intern Med.

[22] Nishiura R, Fujimoto S, Sato Y, Yamada K, Hisanaga S, Hara S, Nakao H, Kitamura K (2009). Elevated osteoprotegerin levels predict cardiovascular events in new hemodialysis patients. Am J Nephrol.

[23] Ozkok A, Caliskan Y, Sakaci T, Erten G, Karahan G, Ozel A, Unsal A, Yildiz A (2012). Osteoprotegerin/RANKL axis and progression of coronary artery calcification in hemodialysis patients. Clin J Am Soc Nephrol.

[24] Sigrist MK, Levin A, Er L, McIntyre CW (2009). Elevated osteoprotegerin is associated with all-cause mortality in CKD stage 4 and 5 patients in addition to vascular calcification. Nephrol Dial Transplant.

[25] Shin JY, Shin YG, Chung CH (2006). Elevated serum osteoprotegerin levels are associated with vascular endothelial dysfunction in type 2 diabetes. Diabetes Care.

[26] Jono S, Ikari Y, Shioi A, Mori K, Miki T, Hara K, Nishizawa Y (2002). Serum osteoprotegerin levels are associated with the presence and severity of coronary artery disease. Circulation.

[27] Montañez-Barragán A, Gómez-Barrera I, Sanchez-Niño MD, Ucero AC, González-Espinoza L, Ortiz A (2014). Osteoprotegerin and kidney disease. J Nephrol.

[28] Kiechl S, Werner P, Knoflach M, Furtner M, Willeit J, Schett G (2006). The osteoprotegerin/RANK/RANKL system: a bone key to vascular disease. Expert Rev Cardiovasc Ther.

[29] Lieb W, Gona P, Larson MG, Massaro JM, Lipinska I, Keaney JF, Rong J, Corey D, Hoffmann U, Fox CS, Vasan RS, Benjamin EJ, O'Donnell CJ, Kathiresan S (2010). Biomarkers of the osteoprotegerin pathway: clinical correlates, subclinical disease, incident cardiovascular disease, and mortality. Arterioscler Thromb Vasc Biol.

[30] Montagnana M, Lippi G, Danese E, Guidi GC (2013). The role of osteoprotegerin in cardiovascular disease. Ann Med.

[31] Koyama S, Tsuruda T, Ideguchi T, Kawagoe J, Onitsuka H, Ishikawa T, Date H, Hatakeyama K, Asada Y, Kato J, Kitamura K (2014). Osteoprotegerin is secreted into the coronary circulation: a possible association with the renin-angiotensin system and cardiac hypertrophy. Horm Metab Res.

[32] Avignon A, Sultan A, Piot C, Mariano-Goulart D (2007). Thuan Dit Dieudonné JF, Cristol JP, Dupuy AM: Osteoprotegerin: a novel independent marker for silent myocardial ischemia in asymptomatic diabetic patients. Diabetes Care.

[33] Omland T, Ueland T, Jansson AM, Persson A, Karlsson T, Smith C, Herlitz J, Aukrust P, Hartford M, Caidahl K (2008). Circulating osteoprotegerin levels and long-term prognosis in patients with acute coronary syndromes. J Am Coll Cardiol.

[34] Kalaycıoğlu E, Gökdeniz T, Aykan AÇ, Hatem E, Gürsoy MO, Ören A, Yaman H, Karadeniz AG, Çelik Ş (2014). Osteoprotegerin is associated with subclinical left ventricular systolic dysfunction in diabetic hypertensive patients: a speckle tracking study. Can J Cardiol.

[35] Kruzliak P, Berezin A, Kremzer A, Samura T, Benacka R, Mozos I, Egom E, Rodrigo L (2016). Global longitudinal strain and strain rate in type two diabetes patients with chronic heart failure: relevance to Osteoprotegerin. Folia Med (Plovdiv).

[36] Kalam K, Otahal P, Marwick TH (2014). Prognostic implications of global LV dysfunction: a systematic review and meta-analysis of global longitudinal strain and ejection fraction. Heart.

[37] Honda H, Qureshi AR, Heimburger O, Barany P, Wang K, Pecoits-Filho R, Stenvinkel P, Lindholm B (2006). Serum albumin, C-reactive protein, interleukin 6, and fetuin a as predictors of malnutrition, cardiovascular disease, and mortality in patients with ESRD. Am J Kidney Dis.

[38] Hermans MM, Brandenburg V, Ketteler M, Kooman JP, van der Sande FM, Boeschoten EW, Leunissen KM, Krediet RT (2007). Dekker FW; Netherlands cooperative study on the adequacy of dialysis (NECOSAD): association of serum fetuin-a levels with mortality in dialysis patients. Kidney Int.

[39] Ciaccio M, Bivona G, Di Sciacca R, Iatrino R, Di Natale E, Li Vecchi M, Bellia C (2008). Changes in serum fetuin-a and inflammatory markers levels in end-stage renal disease (ESRD): effect of a single session haemodialysis. Clin Chem Lab Med.

[40] Hamano T, Matsui I, Mikami S, Tomida K, Fujii N, Imai E, Rakugi H, Isaka Y (2010). Fetuin-mineral complex reflects extraosseus calcification stress in CKD. J Am Soc Nephrol.

