# Supplementary material for: Serum osteoprotegerin in prevalent hemodialysis patients: associations with mortality, atherosclerosis and cardiac function
Source: BMC Nephrol. 2017 Sep 7;18:290. doi: 10.1186/s12882-017-0701-8 (PMC5590161; doi:10.1186/s12882-017-0701-8)
Supplement: Additional file 1: Table S1. — Cardiovascular events and mortality during follow-up. (DOCX 14 kb) [file 12882_2017_701_MOESM1_ESM.docx]

**SUPPLEMENTARY INFORMATION**

**Table S1**. Cardiovascular events (a) and mortality (b) during follow-up

| **a) Cardiovascular events** | 86 (39.1%) |
| --- | --- |
| **Cardiac events** | 56 (65.1%) |
| Acute myocardial infarction | 15 (17.4%) |
| Sudden death | 13 (15.1%) |
| Heart failure | 11 (12.8%) |
| Arrhythmia | 10 (11.6%) |
| Angina | 7 (8.1%) |
| **Non-cardiac vascular disease** | 30 (34.9%) |
| Mesenteric ischemia | 3 (3.5%) |
| Peripheral artery disease | 15 (17.4%) |
| Stroke | 8 (9.3%) |
| Transient Ischemic attack | 4 (4.7%) |
| **b) Overall Mortality** | 74 (33.6%) |
| **Cardiovascular deaths** | 35 (47.3%) |
| Sudden death | 13 (37.1%) |
| Acute myocardial infarction | 8 (22.9%) |
| Stroke | 4 (11.4%) |
| Mesenteric ischemia | 4 (11.4%) |
| Peripheral artery disease | 3 (8.6%) |
| Heart failure | 2 (3%) |
| Arrhythmia | 1 (5.7%) |

.
